# Interaction between socioeconomic deprivation and ethnicity for likelihood of receiving living-donor kidney transplantation

**DOI:** 10.1186/s12882-022-02742-6

**Published:** 2022-03-19

**Authors:** Khalid Khalil, Anna Brotherton, Sue Moore, Felicity Evison, Suzy Gallier, James Hodson, Adnan Sharif

**Affiliations:** 1 East Cheshire NHS Trust, Macclesfield, UK; 2 Department of Nephrology and Transplantation, University Hospitals Birmingham, Edgbaston, Birmingham, B15 2WB UK; 3 Department of Health Informatics, University Hospitals Birmingham, Edgbaston, Birmingham, UK; 4 PIONEER: HDR-UK hub in Acute Care, University of Birmingham, Birmingham, UK; 5 Institute of Translational Medicine, University Hospitals Birmingham, Birmingham, UK; 6 Institute of Immunology and Immunotherapy, University of Birmingham, Birmingham, UK; 7 Department of Nephrology and Transplantation, Queen Elizabeth Hospital, Edgbaston, B15 2WB Birmingham, UK

**Keywords:** Deprivation, Diversity, Ethnicity, Living kidney donors, Kidney transplantation

## Abstract

**Background:**

The interplay between ethnicity and socioeconomic deprivation for living-donor kidney transplantation (LDKT) opportunities is unclear.

**Methods:**

Data for 2040 consecutive kidney-alone transplant recipients receiving an allograft between 1st January 2007 and 30th June 2020 at a single center were retrospectively analyzed. The associations between the proportions of transplants that were LDKT (versus deceased donation) and both ethnicity and socioeconomic deprivation were assessed, with the latter quantified by the Index of Multiple Deprivation (IMD) quintile.

**Results:**

The cohort comprised recipients of White (64.7%), South Asian (21.7%), Black (7.0%) and other (6.6%) ethnic groups. Recipients tended to be from socioeconomically deprived areas, with the most deprived quintile being the most frequently observed (quintile 1: 38.6% of patients); non-White recipients were significantly more likely to live in socioeconomically deprived areas (*p* < 0.001). Overall, 36.5% of transplants were LDKT, with this proportion declining progressively with socioeconomic deprivation, from 50.4 to 27.6% in the least versus most deprived IMD quintile (*p* < 0.001). A significant difference across recipient ethnicities was also observed, with the proportion of LDKTs ranging from 43.2% in White recipients to 17.8% in Black recipients (*p* < 0.001). Both socioeconomic deprivation (*p* < 0.001) and ethnicity (*p* = 0.005) remained significant predictors of LDKT on multivariable analysis, with a significant interaction between these factors also being observed (*p* < 0.001). Further assessment of this interaction effect found that, whilst there was a marked difference in the proportions of transplants that were LDKT between White versus non-White recipients in the most socioeconomically deprived groups (39.5% versus 19.3%), no such difference was seen in the least deprived recipients (48.5% versus 51.9%).

**Conclusions:**

Whilst both socioeconomic deprivation and non-White ethnicity are independent predictors for lower proportions of LDKTs, the significant interaction between the two factors should be appreciated.

## Introduction

For many patients living with end-stage kidney failure, receiving a kidney from a living donor is the best treatment option in terms of survival and quality of life [1]. Despite this being widely acknowledged, rates of living-donor kidney transplantation (LDKT) have stagnated globally, and many barriers have been identified to explain this phenomenon [2]. Two of the most identified barriers to receiving a living-donor kidney are non-White ethnicity and socioeconomic deprivation [3–10]. However, the interaction between these two factors remains poorly understood, and there are conflicting reports in the literature on this issue. For example, Gill and colleagues have observed disparity in the rates of living-donor kidney donation among African-Americans in the United States (US) when comparing higher versus lower income quintiles, suggesting lower rates for African-Americans in the lowest income group [4]. By contrast, Udayaraj and colleagues did not find any significant interaction between socioeconomic status and ethnicity in their population-cohort study in the United Kingdom (UK), suggesting that the effect of socioeconomic deprivation on the uptake of LDKT was similar across all ethnic groups [5].

For transplant centers that encompass large cohorts of both non-White demographics and areas of socioeconomic deprivation, it is important to clearly ascertain the interaction between these two variables, to ensure resources to encourage LDKT are targeted appropriately. Therefore, the aim of this study was to explore the proportions of living versus deceased donation, and explore the possible interaction between ethnicity and socioeconomic deprivation on LDKT, in a large single-center analysis encompassing an ethnically and socioeconomically diverse region of England.

## Materials and methods

### Study population

We undertook a retrospective cohort analysis of all consecutive adult kidney-alone transplants performed at a single-center (University Hospitals Birmingham) between 1st January 2007 and 30th June 2020. Birmingham, and the wider West Midlands territory, is the second most ethnically diverse region in the UK, [11] making it an ideal study population for this analysis. Our only exclusion criterion was transplant of multiple organs; all other kidney allograft recipients were eligible for inclusion. Data were electronically extracted by the Department of Health Informatics for every study recruit, with manual data linkage to additional electronic patient records.

This study is reported according to the RECORD (REporting of studies Conducted using Observational Routinely-collected health Data) statement [12] and the STROBE reporting guidelines [13].

### Definition of ethnicity

We utilized existing pre-determined ethnicity classifications, as obtained from electronic patient records, which were cross-checked against data from the UK Transplant Registry. Ethnicity was classified into the following categories: White, Black, South Asian (also referred to as Indo-Asian) and “other”; patients where this was not recorded were excluded from the analysis of ethnicity.

### Definition of socioeconomic deprivation

Socioeconomic deprivation was assessed based on a recipients’ home address at the time that they underwent transplantation. This was quantified using the Index of Multiple Deprivation (IMD), a multiple deprivation model calculated at the local level area, as utilized by the UK Government [14]. The IMD is a composite construct of seven domains reflective of area’s socioeconomic deprivation, namely: 1) Income Deprivation, 2) Employment Deprivation, 3) Health Deprivation and Disability, 4) Education Skills and Training Deprivation, 5) Barriers to Housing and Services, 6) Living Environment Deprivation, and 7) Crime. The resulting IMDs are then divided into national quintiles, with quintile one being the most deprived, and quintile five the least deprived. For this study, the IMD quintile was treated as if it were a continuous variable for regression modelling. However, for comparisons across groups, the IMD quintiles were combined to form a ‘more deprived’ (quintiles one and two), ‘intermediate’ (quintile three) and ‘less deprived’ (quintiles four and five) group, to maximize the within-group sample sizes.

### Definitions of other variables

Recipient demographics, including age, body mass index (BMI), comorbidities and dialysis status were recorded as of the time of transplantation. The time on the waiting list was defined as starting at the date that a patient was added to the list, and ending at the time that the patient received a transplant for those performed prior to 2018. However, from 2018-onwards, the definition was changed by NHS Blood and Transplant such that the period commenced on the date that a patient started dialysis [15]. For analysis, the data were based on the definition in use at the time that a patient was added to the waiting list.

### Statistical analysis

Initially, a range of patient factors were compared across groups of IMD using Kruskal-Wallis tests for continuous variables, and Chi-square tests for nominal variables. Predictors of receipt of a LDKT were then assessed using univariable binary logistic regression models, with a multivariable model then produced, to identify factors that were independently predictive of receiving a living-donor organ. Prior to this analysis, the goodness of fit of ordinal and continuous factors was assessed using Hosmer-Lemeshow tests, with factors divided into ordinal categories where poor fit was detected. Factors found to be significant on multivariable analysis were then included in separate models alongside the IMD quintile and an interaction term, to identify the effect of any interplay between socioeconomic deprivation and other cohort characteristics.

Continuous variables are summarized as means ± standard deviations (SDs) where approximately normally distributed, with medians and interquartile ranges (IQRs) used otherwise. For univariable analyses, cases with missing data for a factor were excluded from the analysis of the affected factor; multivariable analyses used a complete-cases approach to missing data. All analyses were performed using IBM SPSS 24 (IBM Corp. Armonk, NY), with *p* < 0.05 deemed to be indicative of statistical significance throughout.

### Approvals

This study received institutional approval and was registered as an audit (audit identifier; CARMS-12578). The study was conducted in accordance with the Declaration of Helsinki ethical standards. Formal participant informed consent was not required for this study, as it utilized anonymized pre-existing data from electronic health records. The corresponding author had full access to all data.

## Results

### Cohort characteristics

**Fig. 1:**
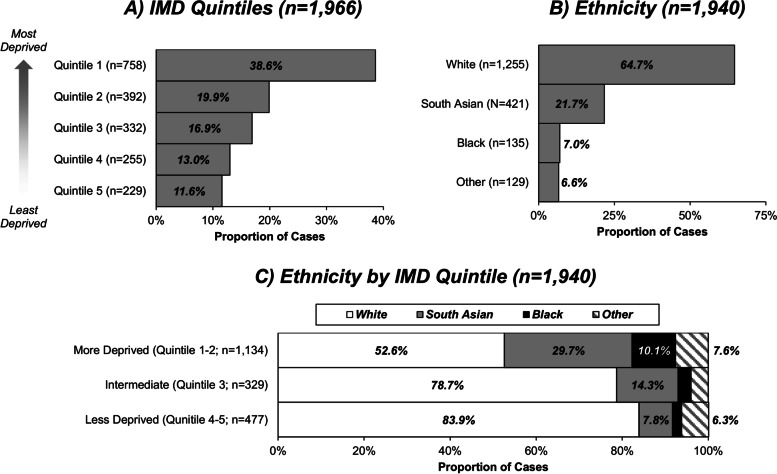
Distributions of IMD and ethnicity. Unlabeled bars each comprise < 5% of the cohort

Data were available for a total of *n* = 2040 transplants. IMD was unavailable in *n* = 74 (3.6%) cases, who were excluded from subsequent analysis. Of the remaining *n* = 1966, IMD quintile 1 (i.e. the most socioeconomically deprived) was the most common (*n* = 758; 38.6%; Fig. 1a). Ethnicity was recorded for *n* = 1940 (98.7%), with the cohort being predominantly of White ethnicity (*n* = 1255; 64.7%; Fig. 1b). The donor type was recorded in *n* = 1962 (99.8%) cases, with organs being from living donors in *n* = 717 (36.5%) transplants. Deceased-donor organs were from donors after brain death (*n* = 798; 40.7%) or donors after cardiac death (*n* = 290; 14.8%), with the type of deceased donor not recorded for the remainder (*n* = 157; 8.0%).

### Associations with IMD score

For initial analysis, the IMD quintiles were combined to form three groups, “More Deprived”, “Intermediate”, and “Less Deprived”. Comparison between these groups found a significant difference in the distribution of ethnicities (*p* < 0.001), as shown in Table 1 and Fig. 1c, with a preponderance of South Asian and Black patients in the more deprived group, comprising 29.7 and 10.1% of cases respectively, compared to 7.8 and 2.1% in the less deprived group. Patients in the more deprived group were also found to be significantly younger (*p* < 0.001), to be more likely to have diabetes (*p* = 0.004), more likely to be on dialysis at the time of transplant (*p* < 0.001), and to have a longer times on the waiting list (*p* = 0.009). Levels of deprivation also varied over the study period (*p* < 0.001), with 54.6% of those transplanted in 2007–2011 being in the more deprived subgroup, compared to 64.0% in 2016–2020.

**Table 1:** Cohort characteristics by IMD quintile

		IMD Quintile			
	N	***More Deprived*** ***(Quintile 1–2)***	***Intermediate*** ***(Quintile 3)***	***Less Deprived*** ***(Quintile 4–5)***	***p***-Value
Age (Years)	1966	46 ± 13	48 ± 14	49 ± 15	**< 0.001**
Gender (% Male)	1966	687 (59.7%)	183 (55.1%)	297 (61.4%)	0.188
Ethnicity	1940				**< 0.001**
*White*		596 (52.6%)	259 (78.7%)	400 (83.9%)	
*South Asian*		337 (29.7%)	47 (14.3%)	37 (7.8%)	
*Black*		115 (10.1%)	10 (3.0%)	10 (2.1%)	
*Other*		86 (7.6%)	13 (4.0%)	30 (6.3%)	
Body Mass Index (kg/m^2^)	1836	27.4 ± 4.7	26.8 ± 4.5	27.0 ± 4.6	0.076
Diabetes	1914	153 (13.7%)	25 (7.8%)	46 (9.7%)	**0.004**
Hypertension	1914	692 (62.0%)	192 (59.6%)	276 (58.0%)	0.298
Previous Transplants	1966	64 (5.6%)	23 (6.9%)	19 (3.9%)	0.162
On Dialysis	1914	758 (67.9%)	192 (59.6%)	260 (54.6%)	**< 0.001**
Time on Waiting List (Months)	1924	30.8 (12.0, 55.8)	25.8 (7.9, 53.7)	24.4 (9.5, 48.2)	**0.009**
Year of Transplant	1966				< 0.001*
*2007–2011*		350 (30.4%)	117 (35.2%)	174 (36.0%)	
*2012–2015*		353 (30.7%)	100 (30.1%)	174 (36.0%)	
*2016–2020*		447 (38.9%)	115 (34.6%)	136 (28.1%)	

Continuous factors are reported as median (interquartile range) or mean ± standard deviation, with p-values from Kruskal-Wallis tests. Categorical factors are reported as N (column %), with p-values from Chi-square tests, unless stated otherwise. Bold p-values are significant at *p* < 0.05. *p-Value from Kruskal-Wallis test, as the factor is ordinal

### Associations with living-donor kidney transplants

**Fig. 2:**
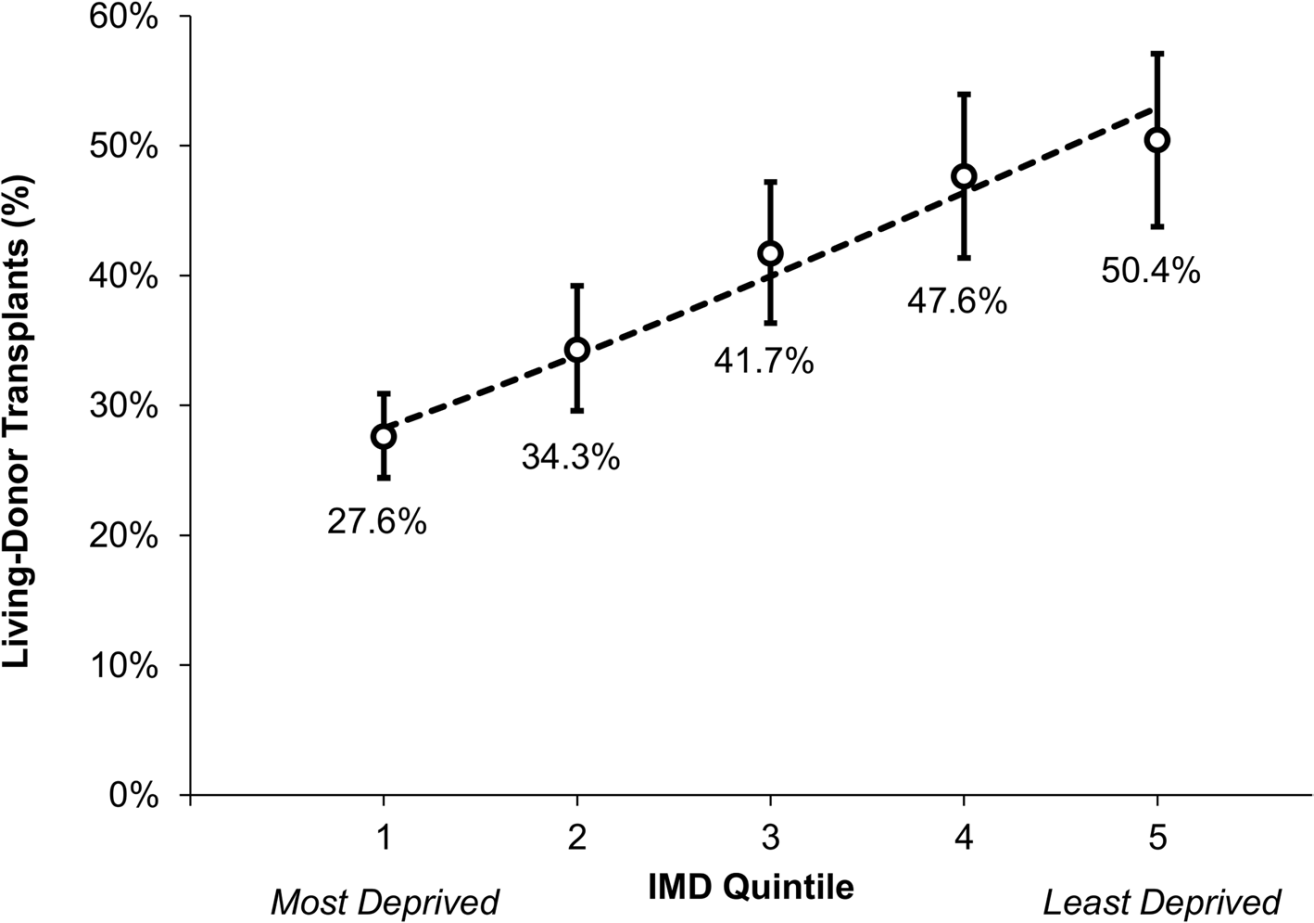
Association between the proportions of living-donor transplants and IMD quintile. Points represent the observed proportions within each IMD quintile, with whiskers representing the 95% confidence intervals. The broken line is from a univariable binary logistic regression model, with the IMD quintile treated as a continuous covariate (as per Table 3)

The proportions of transplants that were LDKT increased progressively with the IMD quintile (Fig. 2, *p* < 0.001), from 27.6% in the most deprived group (IMD quintile 1) to 50.4% in the least deprived group (IMD quintile 5). LDKT proportions also differed significantly with ethnicity (*p* < 0.001), ranging from 43.2% in White recipients to 17.8% in Black recipients. Of the other factors considered (Table 2), older recipients (*p* < 0.001), those with diabetes (*p* = 0.002), those on dialysis at the time of transplant (*p* < 0.001), and those with a longer time on the waiting list (*p* < 0.001) were significantly less likely to receive living-donor kidneys. The proportion of LDKTs also declined significantly over the study period, comprising 44.4% of transplants performed in 2007–2011, compared to 26.4% in 2016–2020 (*p* < 0.001).

**Table 2:** Associations between cohort characteristics and the proportions of transplants of living-donor organs

	Donor Type		
	***Deceased***	***Living***	***p***-Value
IMD Quintile			< 0.001**
*1 (Most Deprived)*	549 (72.4%)	209 (27.6%)	
*2*	257 (65.7%)	134 (34.3%)	
*3*	193 (58.3%)	138 (41.7%)	
*4*	133 (52.4%)	121 (47.6%)	
*5 (Least Deprived)*	113 (49.6%)	115 (50.4%)	
Age (Years)*			< 0.001**
*< 35*	215 (54.6%)	179 (45.4%)	
*35–44*	247 (61.8%)	153 (38.3%)	
*45–54*	328 (64.8%)	178 (35.2%)	
*55–64*	306 (67.5%)	147 (32.5%)	
*65+*	149 (71.3%)	60 (28.7%)	
Gender			0.298
*Female*	516 (64.8%)	280 (35.2%)	
*Male*	729 (62.5%)	437 (37.5%)	
Ethnicity			< 0.001**
*White*	710 (56.8%)	541 (43.2%)	
*South Asian*	329 (78.1%)	92 (21.9%)	
*Black*	111 (82.2%)	24 (17.8%)	
*Other*	77 (59.7%)	52 (40.3%)	
Body Mass Index (kg/m^2^)*			0.076**
*< 18.5*	22 (62.9%)	13 (37.1%)	
*18.5–24.9*	379 (63.6%)	217 (36.4%)	
*25.0–29.9*	409 (59.9%)	274 (40.1%)	
*30.0–34.9*	280 (67.5%)	135 (32.5%)	
*35.0+*	78 (75.7%)	25 (24.3%)	
Diabetes			**0.002**
*No*	1033 (61.2%)	654 (38.8%)	
*Yes*	160 (71.7%)	63 (28.3%)	
Hypertension			0.124
*No*	485 (64.6%)	266 (35.4%)	
*Yes*	708 (61.1%)	451 (38.9%)	
Previous Transplants			0.570
*No*	1175 (63.3%)	681 (36.7%)	
*Yes*	70 (66.0%)	36 (34.0%)	
On Dialysis			**< 0.001**
*No*	332 (47.3%)	370 (52.7%)	
*Yes*	861 (71.3%)	347 (28.7%)	
Time on Waiting List*			< 0.001**
*< 12 Months*	207 (38.8%)	326 (61.2%)	
*12–23 Months*	161 (49.1%)	167 (50.9%)	
*24–35 Months*	181 (68.0%)	85 (32.0%)	
*36–59 Months*	349 (83.5%)	69 (16.5%)	
*60+ Months*	319 (84.6%)	58 (15.4%)	
Year of Transplant*			< 0.001**
*2007–2011*	354 (55.6%)	283 (44.4%)	
*2012–2015*	377 (60.1%)	250 (39.9%)	
*2016–2020*	514 (73.6%)	184 (26.4%)	

Data are reported as N (row %), with p-values from Chi-square tests, unless stated otherwise. Bold p-values are significant at *p* < 0.05. *The factor was divided into categories to illustrate the association with donor type, but p-values were based on the original untransformed variable. **p-Value from Mann-Whitney U test, as the factor is ordinal/continuous

A multivariable analysis was then performed, to identify independent predictors of LDKT. For this analysis, the IMD quintile was treated as a continuous covariate, to account for the ordinal nature of this factor. Of the other continuous variables considered, poor model fit was detected for the time on the waiting list (Hosmer-Lemeshow test: *p* < 0.001), hence this was divided into categories to produce a reliable model. The resulting model found the IMD to be a significant independent predictor of receiving LDKT, with an odds ratio (OR) of 1.23 per quintile (95% confidence interval [CI]: 1.13–1.34, *p* < 0.001, Table 3). Ethnicity was also significant on this analysis (*p* = 0.005), with recipients of both South Asian (OR: 0.65, 95% CI: 0.48–0.89, *p* = 0.007) and Black (OR: 0.54, 95% CI: 0.32–0.90, *p* = 0.018) ethnicity being significantly less likely to receive LDKT, compared to White recipients.

**Table 3:** Multivariable analysis of predictors of living-donor transplant

	Univariable		Multivariable	
	***Odds Ratio (95% CI)***	***p-Value***	***Odds Ratio (95% CI)***	***p-Value***
IMD (per Quintile)	1.30 (1.22–1.39)	**< 0.001**	1.23 (1.13–1.34)	**< 0.001**
Age (per Decade)	0.84 (0.79–0.90)	**< 0.001**	0.95 (0.87–1.04)	0.288
Gender (Male)	1.10 (0.92–1.33)	0.298	1.11 (0.88–1.40)	0.372
Ethnicity		**< 0.001**		**0.005**
*White*	–	–	–	–
*South Asian*	0.37 (0.28–0.47)	**< 0.001**	0.65 (0.48–0.89)	**0.007**
*Black*	0.28 (0.18–0.45)	**< 0.001**	0.54 (0.32–0.90)	**0.018**
*Other*	0.89 (0.61–1.28)	0.522	1.17 (0.75–1.82)	0.487
Body Mass Index (per 5 km/m^2^)	0.90 (0.81–1.00)	**0.044**	0.98 (0.87–1.11)	0.751
Diabetes (Yes)	0.62 (0.46–0.85)	**0.002**	0.67 (0.45–0.98)	**0.041**
Hypertension (Yes)	1.16 (0.96–1.41)	0.124	1.19 (0.94–1.51)	0.146
Previous Transplants (Yes)	0.89 (0.59–1.34)	0.571	1.39 (0.85–2.28)	0.193
On Dialysis (Yes)	0.36 (0.30–0.44)	**< 0.001**	0.61 (0.48–0.78)	**< 0.001**
Time on Waiting List*		**< 0.001**		**< 0.001**
*< 12 Months*	–	–	–	–
*12–23 Months*	0.66 (0.50–0.87)	**0.003**	0.67 (0.49–0.92)	**0.013**
*24–35 Months*	0.30 (0.22–0.41)	**< 0.001**	0.33 (0.24–0.47)	**< 0.001**
*36–59 Months*	0.13 (0.09–0.17)	**< 0.001**	0.13 (0.09–0.19)	**< 0.001**
*60+ Months*	0.12 (0.08–0.16)	**< 0.001**	0.14 (0.09–0.20)	**< 0.001**
Year of Transplant (per Decade)	0.40 (0.32–0.52)	**< 0.001**	0.35 (0.26–0.49)	**< 0.001**

Results of the univariable analysis are from individual binary logistic regression models for each factor. All factors were then entered into a multivariable binary logistic regression model; this analysis was based on *N* = 1724 cases (*N* = 644 events), after exclusion of those with missing data for any of the factors considered. Odds ratios are reported for the stated category relative to the reference category for categorical variables, or per an increase of the stated number of units for ordinal/continuous factors. Bold p-values are significant at *p* < 0.05. *Goodness of fit testing indicated poor model fit when the time on the waiting list was treated as continuous (Hosmer-Lemeshow test: *p* < 0.001), hence it was divided into categories and treated as nominal for analysis

### Interplay between deprivation and other patient characteristics

**Fig. 3:**
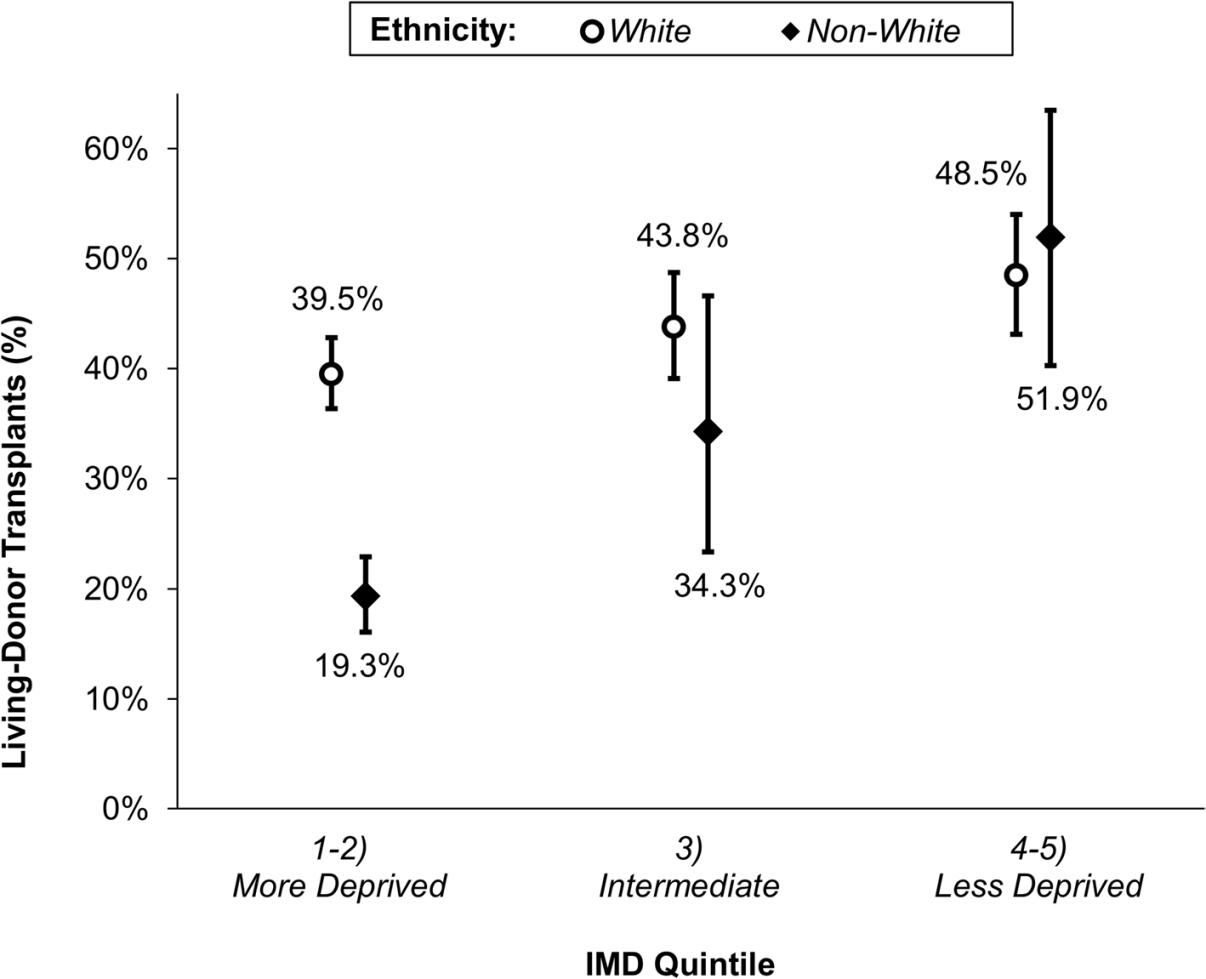
Association between the proportions of living-donor transplants and IMD quintile by ethnicity. Points represent the observed proportions within each combination of IMD quintile and ethnicity, with whiskers representing the 95% confidence intervals. IMD quintiles were combined into three groups, and ethnicity into White versus non-White, in order to maximize within-group sample sizes

Further analysis of the factors identified as significant in the multivariable model found a significant interaction effect between the IMD quintile and ethnicity (*p* < 0.001). Subgroup analysis found the association between IMD and the likelihood of LDKT to be less pronounced in White patients, with an OR of 1.11 per quintile, compared to 1.43–2.02 per quintile in the non-White ethnicities (Table 4). To visualize this effect, the IMD quintiles were first combined to form three groups as previously described, with ethnicity reclassified as White and non-White, in order to maximize the within-group sample sizes (Fig. 3). In the more deprived group, only 19.3% (104/538) of non-White patients received LDKT, compared to 39.5% (235/595) of White patients. However, the magnitude of this difference declined across the IMD quintiles, with LDKTs comprising 51.9% (40/77) of transplants for non-White patients versus 48.5% (193/398) for White patients in the less deprived group.

**Table 4:** Proportions of transplants from living-donors by IMD and ethnicity

IMD Quintile	Ethnicity			
	***White*** ***(N = 1251)***	***South Asian*** ***(N = 421)***	***Black*** ***(N = 135)***	***Other*** ***(N = 129)***
1 (Most Deprived)	138/329 (41.9%)	44/264 (16.7%)	9/95 (9.5%)	15/59 (25.4%)
2	97/266 (36.5%)	20/73 (27.4%)	6/20 (30.0%)	10/27 (37.0%)
3	113/258 (43.8%)	12/47 (25.5%)	4/10 (40.0%)	8/13 (61.5%)
4	103/218 (47.2%)	8/19 (42.1%)	2/5 (40.0%)	7/9 (77.8%)
5 (Least Deprived)	90/180 (50.0%)	8/18 (44.4%)	3/5 (60.0%)	12/21 (57.1%)
Odds Ratio (95% CI)*	1.11 (1.02–1.20)	1.43 (1.18–1.73)	2.02 (1.38–2.96)	1.53 (1.19–1.97)
*p*-Value*	**0.015**	**< 0.001**	**< 0.001**	**< 0.001**

Data are reported as the n/N (%) of patients receiving living-donor organs for each combination of IMD quintile and ethnicity. Bold *p*-values are significant at *p* < 0.05. *Results from binary logistic regression models for each subgroup of ethnicity, with the IMD quintile as a continuous covariate; hence, the odds ratios represent the change in the likelihood of receiving a living-donor organ per increase of one IMD quintile

## Discussion

In this retrospective single-center analysis, we have demonstrated that individuals who are non-White or live in socioeconomically deprived areas have a reduced likelihood of receiving a kidney transplant from a living-donor. Further analysis identified interplay between socioeconomic deprivation and ethnicity such that, whilst individuals of non-White ethnicity had a considerably lower chance of LDKT than their White counterparts in areas with high levels of deprivation, no such effect of ethnicity was observed in areas of low deprivation. Our study confirms the importance of ethnicity and socioeconomic deprivation as barriers to receiving LDKT, and introduces the important interaction between the two. While this study highlights the importance of encouraging discussions of the benefits of LDKT to individuals who live in socioeconomically deprived areas, it suggests appropriate tailoring of information is required for ethnic minority communities living in deprived areas, in order to achieve equity.

Non-White ethnicity and socioeconomic deprivation have long been associated with access to transplantation. For example, in a study of patients starting renal replacement therapy in the UK, Udayaraj and colleagues found those in the most deprived quintile (hazard ratio [HR]: 0.60, 95% CI: 0.54–0.68, *p* < 0.001) and of non-White ethnicity (HR = 0.89 for Black, 0.91 for South Asian) to be significantly less likely to waitlisted for a deceased donor kidney transplant [16]. Where patients go on to receive a transplant, our study suggests that both ethnicity and socioeconomic deprivation may influence the type of organ received. This finding is consistent with a population cohort analysis of registry data from the US performed by Reed and colleagues, who found a composite index of health and socioeconomic status factors to be negatively associated with LDKT, rates of which were 7.3 percentage points lower (95% CI − 12.2 to − 2.3, *p* = 0.004) in areas with greater burden of medical co-morbidity and more socioeconomic deprivation [17]. The study also observed lower rates of LDKT among transplant centers with higher prevalence of ethnic minorities, with rates 7.1 percentage points lower (95% CI − 11.8 to − 2.3, *p* = 0.004) [17]. However, the study did not assess the potential interaction between these two factors.

A recent study that did assess the interaction between socioeconomic status and ethnicity was a multi-center, cross-sectional study of a US cohort by Killian and colleagues. They quantified social deprivation using a social vulnerability index, and found both this and non-White ethnicity to be independently associated with a lower likelihood of LDKT [18]. However, they additional assessed the interaction between these factors and, like our study, found this effect to be significant, such that the disparity in LDKT between Black and White recipients increased with greater community-level vulnerability (ratio of adjusted Relative Risk, 0.67; 95% CI, 0.51–0.87; *p* = 0.003).

A similar interaction effect has also been observed in a study assessing living kidney donation by Gill and colleagues. They analyzed of data from the United Network for Organ Sharing (UNOS) registry, and found higher overall rates of living kidney donation among the African-American versus White population (incidence rate ratio 1.20, 95% confidence interval 1.17–1.24) [4]. However, this effect was mediated by income (a component of socioeconomic status), with the incidence of living kidney donation being lower among African-Americans (vs. White population) in the lowest income quintile, but better among the higher three income quintiles [4]. Their conclusion was that racial disparities associated with living kidney donation are likely related to socioeconomic factors, rather than socio-cultural factors. Our data, exploring this from a recipient perspective, corroborates these findings, and confirms a significant interaction between socioeconomic status and ethnicity for a recipient’s likelihood of receiving a LDKT.

However, reliance on data from the US may not be directly translatable to countries like the UK. Living donors in the UK do not receive financial remuneration for their donation, but are fully reimbursed for their expenses and loss of earnings during their post-operative period. Therefore, financial pressures that may dissuade potential ethnic minority living kidney donors in the US may not be translatable to other countries with universal health coverage. In the UK, Udayaraj and colleagues explored data on 12,282 kidney transplant recipients between 1997 and 2004 [5]. They observed a reduced probability of receiving a kidney from a living donor for ethnic minority recipients, and those residing in socioeconomically deprived areas, with some attenuation of the ethnic differences in their adjusted models, after controlling for socioeconomic status. However, no significant interaction was observed between ethnicity and socioeconomic status, suggesting the effect of socioeconomic status on uptake of living kidney donation is similar across all ethnic groups. This contrasts with our analysis, which found a lower likelihood of LDKT for ethnic minority recipients if they reside in areas of socioeconomic deprivation, with significant statistical interaction between the IMD and ethnicity. However, data from Wu and colleagues, analyzing data from the multi-center Access to Transplantation and Transplant Outcomes (ATTOM) study, confirms our findings of non-White ethnicity and parameters of socioeconomic deprivations to be independent factors predictive of reduced likelihood of receiving a kidney from a living kidney donor [3]. Conversely, a publication from Scotland reported no difference in the proportion of patients receiving a living-donor kidney across the quartiles of the Scottish IMD score [19].

The reasons behind the observed differences across groups of socioeconomic deprivation are speculative, and qualitative research is underway to address this discrepancy. A study conducted by Bailey and colleagues interviewed recipients who had received a deceased donor kidney, and explored the main barriers to LDKT [20]. Socioeconomically deprived recipients often reported a one-sided passive relationship with their clinician, lack of involvement in decision-making, and a lack of knowledge of the available options as some of the main reasons why they did not pursue the option of LDKT [20]. While this work suggests these patients may benefit from more targeted education about their options for kidney transplantation, it also raises concerns about socio-cultural and psychological factors that may hinder discussions in relation to living kidney donation. Ethnic minority individuals appear more likely to consider becoming living kidney donors, with higher rates of living kidney donation per million population than other ethnicities, according to national registry data in the UK [21]. This suggests transplant professionals are attaining some success in getting the message across with regards to the benefits of living kidney donation to ethnic minority communities. Our data adds to the literature by suggesting ethnic minority individuals residing in areas of socioeconomic deprivation are a high-risk group with regards to attaining a LDKT, and require more targeted focus.

Whilst we and others have reported the likelihood of receiving a living kidney donor to be lower for ethnic minority individuals or those resident in areas of socioeconomic deprivation, we do not present any data on the number of living kidney donor candidates who come forward, but fail the assessment. It is plausible that potential living kidney donors from ethnic minority communities and/or poor socioeconomic status come forward in equal numbers, but are ruled out on medical grounds. It is well known that there is a greater burden of health issues such as diabetes and/or hypertension in ethnic minority individuals and for residents within deprived areas [22–24], which could be an important factor, and requires further investigation. Bailey and colleagues have observed potential living kidney donor candidates from ethnic minority communities have a nearly three-fold increased odds of withdrawing from the assessment process (OR: 2.98; 95% CI 1.05–8.44, *p* = 0.04) [25]. Although individuals from most socioeconomically deprived areas appeared to have reduced likelihood of donation, they found the trend with deprivation to be non-linear and consistent with chance (OR per IMD quintile increase: 0.88; 95% CI, 0.75–1.03; *p* = 0.12). In the donor and recipient sex-adjusted analysis, they found the most deprived potential donors to remain the least likely to donate, but this did not persist after adjustment for possible mediators of socioeconomic deprivation on living donation, with most IMD quintiles showing attenuation of the effect estimates. In addition, they reported that people donating to more deprived recipients were more likely to withdraw, but this association was not statistically significant at the 5% level after adjustment for donor age (OR of withdrawal per unit increase in IMD quintile: 1.13; 95% CI, 0.95–1.34; *p* = 0.17).

Considering our findings, and a review of the literature, we suggest educational resources to encourage living kidney donation must reflect the identified obstacles and be tailored to the individual. Strategies could include home-based discussions with allied healthcare professionals, which provide educational advice in a familiar environment, but also engages family and social support networks to aid decision-making for the potential kidney transplant candidate [2]. Importantly, such clinical trials have been shown to boost living kidney donor evaluation by over 50% [26–28]. Streamlining the process for potential living kidney donors, and removing financial disincentives in some countries, are also important interventions to encourage more potential living kidney donors to come forward, or encourage them not to withdraw from the assessment process once started [29, 30]. The overwhelming financial benefits of successful LDKT compared to dialysis means healthcare providers have an incentive to facilitate such pathways, to ensure living kidney work-ups are streamlined and efficient for the benefit of potential candidates. Further research is clearly warranted to ensure we minimize the risk of willing potential donors withdrawing from the work-up pathway for reasons which could be amenable to intervention [31].

The limitations of this study should be appreciated for the correct interpretation of our analysis. The primary limitation was that the study only considered those patients that had received a transplant. As such, those that died on the waiting list, or that were still on the waiting list at the time of data collection will have been excluded. This may have introduced selection bias, particularly if either ethnicity or socioeconomic deprivation influence the likelihood of receiving a transplant, or the time on the waiting list. This is an important limitation which should be rectified for any future work in this area. Secondly, as a single-center study, we were not able to assess any region-specific effects; therefore the findings may not be generalizable to other centers. Thirdly, the IMD was calculated based on the recipient’s home address at the time of kidney transplant surgery. However, it is possible that patients may have changed residence during their time on the waitlist, potentially multiple times in those with long waits. Finally, the study was retrospective in nature, and so prone to all the shortcomings of such analyses, including the inability to establish causation, and the potential effects of unmeasured or intangible confounders. On the latter point, there were some variables for which data were not available, but which may have a significant influence on LDKT opportunities, including marital status and the number of siblings. There was also some granular data that were not available, such as time since dialysis commencement. The UK allocation system also evolved during this time, fundamentally after 2018 when waiting time was calculated from commencement of dialysis rather than time joining the waiting list. This confounder will impact upon the granular data for waiting time versus dialysis status.

Whilst the results of the study should be generalizable to other centers within the UK, they may not be applicable in other countries for several reasons. Firstly, the quantification of socioeconomic deprivation by the IMD quintile may not translate well to other countries, where either the baseline level of deprivation, or disparity between the most and least deprived residents, is different to that in the UK. The findings may also not be applicable to cultures with different views on the ethics or appropriateness of living-donor transplantation, particularly if these vary by ethnicity. Finally, the UK does not offer financial incentives for becoming a living donor, but does reimburse donors for their expenses. As such, the results of the analysis may not be generalizable to countries that use a different system of financial remuneration, particularly with respect to the effect of socioeconomic deprivation.

To conclude, our study identifies both socioeconomic deprivation and non-White ethnicity are being associated with a lower proportion of transplants being LDKT at a patient-level, with the effect of ethnicity being most pronounced in areas with the greatest levels of socioeconomic deprivation. This analysis highlights the importance of encouraging discussions relating to the benefits of LDKT to individuals who reside in socioeconomically deprived areas. However, it also suggests ethnic minority candidates in these deprived areas require targeted intervention to maximize opportunities to facilitate receiving a LDKT.

## Data Availability

The data that support the findings of this study are not publicly available due to legal restrictions but available from the corresponding author on reasonable request.

## Abbreviations

IMD: Index of Multiple Deprivation
LDKT: Living-donor kidney transplantation
